# Editorial: Epigenetics and molecular genetics of rare tumors

**DOI:** 10.3389/fonc.2022.1049140

**Published:** 2022-11-03

**Authors:** Mauro Tognon, George A. Calin, Luis Del Valle

**Affiliations:** ^1^ Department of Medicine, Laboratories of Cell Biology and Molecular Genetics, Section of Experimental Medicine, Department of Medical Sciences, University of Ferrara, Ferrara, Italy; ^2^ Department of Medicine, MD Anderson Cancer Center, University of Texas, Houston, TX, United States; ^3^ Department of Medicine, Stanley S. Scott Cancer Center, Louisiana State University Health, New Orleans, LA, United States; ^4^ Department of Pathology, Louisiana State University Health, New Orleans, LA, United States

**Keywords:** epigenetics, molecular genetics, rare tumor, DNA methylation, histone (de)acetylation, microRNA

In recent years, many reports have been published on the epigenetics and molecular genetics of rare tumors. Indeed, the advent of new techniques has enabled dysregulated microRNA, DNA methylation and histone modifications to be analyzed in rare tumors with different histotypes.

In this special issue, dedicated to the “Epigenetics and Molecular Genetics of Rare Tumors”, some rare tumor epigenetic and molecular genetic profiles were investigated, and the state of the art was reviewed by various authors. Significant data were obtained on basic topics relating to some rare cancers.

In their review, Mazziotta et al. reported on “*The Role of Histone Post-Translational Modifications in Merkel Cell Carcinoma*”. Interestingly, histone post-translational modifications (PTMs) in Merkel cell carcinoma (MCC) emerged as potential diagnostic and prognostic markers, with consequent therapeutic implications for this deadly tumor.

In another review, Gallenga et al. reported on the state of the art of uveal melanoma (UV) metastasis. UM patients are known to develop metastases from micrometastases, which are undetectable at diagnosis. New data on cytogenetic prognostic markers and biochemical pathways indicate a correlation to UM metastasis development. It emerged that genetic analysis is crucial for metastatic risk prediction, as well as for patient management and follow-up.

Moreno et al. reviewed the available literature on nuclear protein in testis (NUT) carcinoma, which is a rare, highly aggressive, poorly differentiated carcinoma occurring mostly in adolescents and young adults. Insight into the role of multiple *NUTM1* gene rearrangements in carcinogenesis and its impact on underlying molecular mechanisms may result in the development of novel targeted therapies for this deadly cancer.

In her review, Crabtree reports on epigenetic regulation in gastroenteropancreatic neuroendocrine tumors. Epigenetic regulation in these rare cancers includes DNA methylation, chromatin remodeling and regulation by non-coding RNAs. Epigenetic signatures may serve as biomarkers and innovative targets in both pharmacological and theranostic approaches to new treatment development.

In their investigation, Julius et al. studied ocular surface squamous neoplasia (OSSN). It turned out that OSSN onset seems to be linked to Epstein-Barr Virus (EBV) infection, which is a DNA tumor virus. Other oncogenic viruses, including HPV, tested negative for their potential role on OSSN development.

The study by Mao et al. investigated lung adenocarcinoma (LUAD). Methylated RNA immunoprecipitation sequencing (MeRIP-seq) and RNA sequencing (RNA-seq) were used to identify differential N6-methyladenosine (m6A) modifications between tumor and tumor-adjacent normal tissues. Since m6A modification seems to play a key role in tumor progression, LUAD transcriptome-wide analysis was undertaken. Data indicate that m6A modifications play an important role in the progression and prognosis of LUAD, suggesting that m6A may become a potential new therapeutic target for patients with this tumor.

Pantaleo et al. analyzed *SDHA* germline variants in adult patients with SDHA-mutant gastrointestinal stromal tumor (GIST). DNA sequencing data indicate that SDHA pathogenic germline variants are highly frequent in *SDHA*-deficient GIST cases in both young people and older adult patients, too. Genetic testing and surveillance of SDHA mutation carriers and relatives are proposed, together with accurate clinical follow-up.

Zhao et al. studied ocular adnexal mucosa-associated lymphoid tissue lymphoma (OAML) by whole-exome sequencing. Novel *IGLL5* gene mutations, alongside *MSH6, DIS3, FAT1*, and *TMEM127* gene mutations, were detected. *IGLL5* appeared as recurrently mutated in 24% of OAML tumors and associated with a higher recurrence rate. These new data suggest that additional lymphomagenesis pathways in OAML may exist.

This Research Topic deals with the current state-of-the art of some rare tumors. Recent studies with epigenetic and molecular genetic approaches have enabled our knowledge of rare tumors to be improved, with new data attracting the scientific interest of laboratory and clinic investigators. At the same time, biotech companies and pharmaceutical firms have elaborated and processed new diagnostic tools and therapeutic drugs to identify and cure rare cancers. New molecular targets, detected in rare tumors, may allow novel clinical trials to be set up for patient treatment and innovative technical approaches for diagnosis. These innovations may help to improve the treatment of rare cancers significantly.

The three editors, Tognon, Calin and Del Valle are indebted to all the authors that contributed to this significant Research Topic with their reviews and articles dedicated to the epigenetics and molecular genetics of rare tumors. We believe that works on this Research Topic will trigger new investigations into rare tumors, which in turn will help to develop innovative treatments for these deadly diseases with positive outcomes for oncological patients.

## Author contributions

All authors listed have made a substantial, direct, and intellectual contribution to the work and approved it for publication.

## Funding

The investigations of Mauro Tognon have been supported by the Associazione Italiana per la Ricerca sul Cancro (AIRC), grant IG 21617.

## Acknowledgments

We thank Prof. Georgia Emma Gili for revising the English text of the manuscript.

## Conflict of interest

The authors declare that the research was conducted in the absence of any commercial or financial relationships that could be construed as a potential conflict of interest.

## Publisher’s note

All claims expressed in this article are solely those of the authors and do not necessarily represent those of their affiliated organizations, or those of the publisher, the editors and the reviewers. Any product that may be evaluated in this article, or claim that may be made by its manufacturer, is not guaranteed or endorsed by the publisher.

